# Strategies to Increase On-Target and Reduce Off-Target Effects of the CRISPR/Cas9 System in Plants

**DOI:** 10.3390/ijms20153719

**Published:** 2019-07-30

**Authors:** Zahra Hajiahmadi, Ali Movahedi, Hui Wei, Dawei Li, Yasin Orooji, Honghua Ruan, Qiang Zhuge

**Affiliations:** 1 Co-Innovation Center for Sustainable Forestry in Southern China, Key Laboratory of Forest Genetics & Biotechnology, Ministry of Education, Nanjing Forestry University, Nanjing 210037, China; 2 Department of Biotechnology, Faculty of Agricultural Sciences, University of Guilan, Rasht 4199613776, Iran; 3 College of Materials Science and Engineering, Nanjing Forestry University, No. 159, Longpan Road, Nanjing 210037, China

**Keywords:** Cas9, CRISPR, off-target effect, on-target mutation, aptazyme

## Abstract

The CRISPR/Cas9 system (clustered regularly interspaced short palindromic repeat-associated protein 9) is a powerful genome-editing tool in animals, plants, and humans. This system has some advantages, such as a high on-target mutation rate (targeting efficiency), less cost, simplicity, and high-efficiency multiplex loci editing, over conventional genome editing tools, including meganucleases, transcription activator-like effector nucleases (TALENs), and zinc finger nucleases (ZFNs). One of the crucial shortcomings of this system is unwanted mutations at off-target sites. We summarize and discuss different approaches, such as dCas9 and Cas9 paired nickase, to decrease the off-target effects in plants. According to studies, the most effective method to reduce unintended mutations is the use of ligand-dependent ribozymes called aptazymes. The single guide RNA (sgRNA)/ligand-dependent aptazyme strategy has helped researchers avoid unwanted mutations in human cells and can be used in plants as an alternative method to dramatically decrease the frequency of off-target mutations. We hope our concept provides a new, simple, and fast gene transformation and genome-editing approach, with advantages including reduced time and energy consumption, the avoidance of unwanted mutations, increased frequency of on-target changes, and no need for external forces or expensive equipment.

## 1. Introduction

Plant biotechnology has entered a new era of mutagenesis methods that can replace conventional mutagenesis approaches such as EMS (Ethyl methanesulfonate) and γ-radiation. The CRISPR/Cas9 (clustered regularly interspaced short palindromic repeat/CRISPR associated protein 9) system was first found in bacterial species, and has been used as a powerful genome editing tool since 2013 [1]. The abovementioned system can lead to direct mutagenesis of the gene of interest [2]. The CRISPR/Cas9 system requires two components, single guide RNA (sgRNA) and Cas9, to recognize and cleave the target DNA, thereby instigating the DNA repair mechanism and leading to gene mutation. Cas9 contains two domains: (1) an HNH domain, and (2) a Ruc-V-like domain. The first domain cleaves the complementary strand of CRISPR RNA (crRNA), and the second domain breaks the opposite strand of the double-strand DNA. The sgRNA consists of the seed sequence and nonseed sequence with a length of ~100 bp. A 20 bp region at the 5′-end of the sgRNA helps to identify the target sequence adjacent to the protospacer adjacent motif (PAM) sequence, and is the most important part of the guide RNA (gRNA) sequence in the CRISPR/Cas9 system [3]. The loop structure at the 3′-end of the sgRNA can be recognized by Cas9, and the Cas9/sgRNA complex cleaves the target DNA and forms a double-stranded break (DSB). A DSB induces a DNA repair mechanism, such as nonhomologous end joining (NHEJ) or homology-directed repair (HDR). Usually, a DSB is repaired by NHEJ, which leads to mismatches (knock-out). If a homologous DNA sequence (Donor DNA) is present, HDR results in foreign gene replacement (knock-in) [4,5]. Therefore, CRISPR/Cas9 can generate targeted changes in the plant genome. One of the important advantages of the CRISPR/Cas9 system compared with other conventional genome editing tools, such as zinc finger nucleases (ZFNs) and transcription activator-like effector nucleases (TALENs), is that multiple sgRNAs can be introduced simultaneously into cells to create multiplex targeting mutations [6]. Likewise, the CRISPR/Cas9 system is simple and affordable compared with ZFNs and TALENs [2,7]. To date, more than 25 plant species (~100 genes) have been edited successfully using the CRISPR/Cas9 system (Table 1) [7]. However, a serious concern about CRISPR/Cas9 is unwanted (off-target) mutations. The targeting specificity of Cas9 depends on the seed sequence and the presence of the PAM adjacent to the target sequence, but off-target mutations can occur in the genome with as many as three to five mismatches within the PAM-distal region of the target [8,9]. In most plant species, off-target mutations are less important and can be eliminated by back-crossing [10]. This review describes off-target detection methods and strategies for minimizing off-target effects in plants.

## 2. Off-Target Detection Methods

The specificity of CRISPR/Cas9 is affected by various factors, such as the Cas9/sgRNA complex concentration and features of the off-target sites [11]. Off-target mutation is a crucial concern regarding the development of the CRISPR/Cas9 system in plants. The frequency of undesirable mutations has been reported to be low in many plants [12,13]. Therefore, CRISPR/Cas9 is more specific in plants than in human cells [13,14]. Feng et al. (2014) and Peterson et al. (2016) evaluated CRISPR/Cas9 specificity in *Arabidopsis thaliana* using whole genome sequencing (WGS) and deep sequencing, respectively. Based on their results, CRISPR/Cas9 is highly specific in plants due to low expression levels of the Cas9 protein that lead to undetectable levels of off-target mutations [14,15]. In most CRISPR/Cas9 studies in plants, a low frequency of off-target mutation has been reported, which might be due to its occurrence in noncoding regions and, thus, the inability to detect off-target effects in the phenotype [16]. Zhang et al. (2018) showed a high frequency of undesirable mutations induced by CRISPR/Cas9 in *A. thaliana,* despite the use of high-specificity sgRNA. Their results revealed that off-target effects can be aggravated in the second generation (17% in the first generation vs. 85% in the next generation) [11]. Accurate identification of possible off-target sites is an important challenge in the field of genome editing. In higher plants, undesirable mutations resulting from the CRISPR/Cas9 system are generally rare and can be detected by WGS. Unwanted mutations can also be avoided using more specific sgRNAs [17,18,19]. A few methods in the literature for the prediction of off-target sites in plants can be found [20]. Different bioinformatics tools have been introduced to design highly specific sgRNAs with minimal off-target activity. One of the main prediction tools for off-target mutations in plants is CRISPR-PLANT v2 [21]. In this online software, global and local alignment results have been combined with NGG and NAG spacer sequences to assess the unwanted mutation probability. This software can reveal the highest sensitivity among all off-target prediction tools and can be used in seven plant genomes, including *Arabidopsis thaliana*, *Brachypodium distachyon*, *Oryza sativa*, *Medicago truncatula*, *Glycine max*, *Solanum lycopersicum,* and *Sorghum bicolor* [21]. Several approaches for off-target detection, including deep sequencing and online prediction software, have been introduced in eukaryotes [22,23,24]. However, although in silico and in vitro methods for the investigation of potential off-target sites have been developed, it is difficult to obtain exact predictions of the occurrence of unwanted mutations in vivo [25]. For instance, Jia et al. (2014) investigated potential unwanted mutations using BLASTn tool and they identified 46 off-target sequences. However, no off-target mutations were detected in their experiments. In another study, Fister et al. (2018) applied Geneious CRISPR site tool to predict off-target mutagenesis. Based on their in silico analysis, nine unintended mutations could occur while no off-target changes have been observed in their experiments. Taken together, further studies are necessary to develop a precision off-target prediction system.

## 3. Strategies for Reducing Off-Target Mutations

### 3.1. The Length, Mismatches, and GC Contents of Guide RNAs

The CRISPR/Cas9 system has been used for various purposes, such as loss of gene expression (knock-out), expression of a new gene (knock-in), and increased or decreased expression of a gene of interest. The CRISPR/Cas9 system has been used successfully in various plants, including rice, tomato, *A. thaliana*, maize, poplar, grape, cucumber, and petunia [47,60,61,62,63]. Prediction and avoidance of off-target mutations are necessary for functional genome analysis, especially in the breeding of plants where the whole-genome sequence is still lacking [64]. Studies on CRISPR/Cas9 in plants have shown that the most important point in increasing on-target mutagenesis is designing highly specific sgRNA, which can lead to undetectable off-target changes [14,15,65]. Unwanted mutagenesis can occur due to the similarity of the sgRNA to unintended sites. Therefore, to minimize off-target mutations, it is essential to avoid using sgRNAs containing seeds that are homologous to many other genome loci [66]. Carefully designed sgRNAs can facilitate specific targeting, even if many homologous loci exist in the studied genome [32]. A GC content of the target sequence higher than 70% may also increase off-target effects [67]. Consistent with these findings, Li et al. (2016) found that GC content between 65–80% can increase unexpected mutations. However, sgRNAs with GC content greater than 50% are efficient enough to increase on-target mutagenesis due to strong binding to the target sites [15,51]. Ren et al. (2014) found a positive correlation between mutagenesis and the GC content of the PAM-proximal part of the sgRNAs [68]. Due to the low frequency of off-target mutations compared to on-target mutations, recovery of the on-target mutations can occur in all experiments [69]. The specificity of the sgRNA is mainly dependent on the seed sequence (10–12 bp 5′ proximal of the PAM) [8]. The use of sgRNA with a partial mismatch out of seed sequence leads to off-target changes. Therefore, specific design of the sgRNA decreases unintended mutation, which has been reported in many plants, such as *A. thaliana* [14] and *O. sativa* [15]. Likewise, the length of sgRNA can affect the frequency of unwanted mutations. Sugano et al. (2018) appraised the effect of sgRNA lengths (16, 17, 18, and 20 bp) on the efficiency of genome-editing and frequency of off-target mutations in *Marchantia polymorpha*. According to their results, the sgRNA with a length up to 17 nucleotides (16 and 17 bp) and more than 17 nucleotides (18 and 20 bp) revealed low and higher genome-editing efficiency, respectively. They did not observe any unwanted mutations using 20 bp-sgRNA [70]. In contrast, Fu et al. (2014) ascertained that sgRNA with a length of 17 bp revealed low unintended changes in mammalian cells [71]. Taken collectively, it is better to use 18-bp sgRNA to reduce the frequency of off-target mutations. Furthermore, chimeric guide RNA with partial DNA replacement strategy has been used to decrease off-target effects in human cells [72]. Yin et al. (2018) ascertained that crRNAs with partial DNA nucleotides at their 5′ end could significantly decrease unwanted changes due to less tolerance to mismatches of DNA-DNA compared to DNA-RNA duplexes. The use of multiple sgRNAs for a target gene also increases the mutation frequency in rice and tomato plants [19,52,69,73]. The ideal sgRNA leads to maximum on-target and minimum off-target mutagenesis. Lin et al. (2014) suggested some sgRNA design guidelines to reduce the potential off-target effects in human cells, which can also be helpful for plants: (1) Avoid target sequences with more than three mismatches within 7–10 bp of the PAM; and (2) avoid sgRNA bulges within 12 bp of the PAM [74]. Generally, the frequency of off-target mutations in plants is less than the frequency of somatic mutations during tissue culture, due to the possibility of minimizing the frequency of unwanted changes in the CRISPR system [75]. For instance, Li et al. (2016) evaluated off-target mutations using four sgRNAs in rice plants. Based on their results, the highest unwanted changes (67.5%) were observed when there were two mismatched bases between sgRNA and its off-target site, while the lowest off-target rate (2.5%) was found for sgRNA with six mismatches. Therefore, using sgRNAs with more than two mismatches with an off-target site can avoid unintended changes in rice [76].

### 3.2. Concentration of sgRNA/Cas9

Another strategy for decreasing off-target effects is a low concentration of the sgRNA/Cas9 complex [77]. Therefore, the selection of promoters is necessary to control the expression of Cas9. In *A. thaliana*, Cas9 under the control of the constitutive CaMV35S promoter revealed a low editing frequency compared to Cas9 under the control of an egg-cell specific promoter (ECS) [33]. This could be due to the stronger expression of Cas9 by using ECS promoter compared to CaMV35S that cause high-efficiency of CRISPR/Cas9 genome editing system in tested plants. Likewise, The use of embryo-specific promoters such as YAO led to efficient genome editing in *Citrus sinesis* by increasing the expression of Cas9 and sgRNA during plant reproduction [78]. Based on the results of other studies, a high level of sgRNA leads to low editing potential of the CRISPR/Cas9 system in tomato and *A. thaliana* [51,79]. In monocots, using plant endogenous promoter to express Cas9 leads to higher on-target mutations than CaMV35S [18,66,80,81]. Likewise, Sun et al. (2015) investigated the efficiency of soybean U6–10 and *Arabidopsis* Ubi (AtUbi) promoters for targeted gene mutagenesis in *G. max*. Based on their findings, the frequency of on-target mutations can increase 2–4-fold when the endogenous U6-10 is used compared to the AtUbi [82]. However, the expression of Cas9 driven by AtUbi and 2 × CaMV35S promoters causes similar efficiency of on-target changes in tomato plants [51]. A number of studies have been conducted to increase the heritability of mutations using specific promoters as well [83,84]. They reported that using meristem-specific promoters such as CLAVATA3, APETARA1, and INCURVATA2 can lead to an increase in the heritability and efficiency of mutations. According to the results of Xu et al. (2014), T1 rice plants containing both Cas9 and sgRNA showed unwanted mutations, while unintended mutations were not observed in plants carrying only one of those genes. It can be concluded that by selecting suitable T1 plants in which the CRISPR components have been segregated, off-target mutations might be avoided [33]. In conclusion, the selection of promoter depends on the purpose of the research and the target plant species. Likewise, various ratios of sgRNA/Cas9 have been assessed to increase and decrease the frequency of on-target and off-target changes, respectively. Li et al. (2013) investigated the effect of different ratios of sgRNA/Cas9 (1:1, 19:1, and 20:1) on mutation frequency in *A. thaliana.* Based on their results, optimal mutagenesis of targeted *AtPDS3* (5.6%) and *AtFLS2* (1.1%) genes occur only at the mass ratio of 1:1 [26]. Two methods, including DNA ratio of Cas9/cgRNA (chimeric guide RNAs) at 1:1 and co-delivery of Cas9 and cgRNA, have been compared to increase mutagenesis efficiency in *Nicotiana benthamiana* as well [17]. In this case, the mutation frequency of the co-delivery of Cas9 and sgRNA and the mixed ratio of Cas9/cgRNA (1:1) were 12.7% and 1.8%, respectively [17]. Therefore, the existence of Cas9 and cgRNA in a single plasmid construct and co-delivery of them is much more efficient than the ratio of Cas9/cgRNA at 1:1. Malnoy et al. (2016) determined the best ratio of Cas9/sgRNA to achieve maximum mutation efficiency in apple and grape. According to their findings, the 3:1 ratio for *DIPM1* gene (6.7%), the 1:1 and 3:1 ratio for *DIPM2* gene (3.3%), and the 1:1 ratio for *DIMP4* gene (6.9%) have been obtained in apple plants, while the best ratio for *MLO-7* gene in grapevine was 3:1 (0.1%) [46]. Therefore, the efficiency of this strategy to improve the on-target mutagenesis depends on the plant species and the targeted gene.

### 3.3. Transformation Methods

The selection of the transformation method is another important factor in increasing the on-target accuracy. In addition to the common *Agrobacterium*-mediated transformation method, Ali et al. (2015) applied a virus-mediated genome editing system in *N. benthamiana* plants. They used an RNA-virus-based vector named *Tobacco rattle virus* (TRV) to deliver sgRNAs into Cas9-overexpressing *N. benthamiana* plants via agroinfiltration. One of the important advantages of this system is the development of transgene-free plants because they do not integrate into the targeted plant’s genome [85]. Although being time-consuming is an important shortcoming of the *Agrobacterium*-mediated genome editing system, the TRV-mediated editing system is fast, convenient, and can systematically infect targeted plants. Due to the lack of inheritance of mutations in the TRV-based guide RNA delivery system (less than 15%), further research to develop the virus-based genome editing system is necessary to transmit on-target changes to the next generation. Likewise, delivery of the sgRNA and nuclease as RNP complexes via electroporation into plant protoplasts has demonstrated a low frequency of off-target mutations. The Cas9-sgRNA complexes cleaved the target site and degraded rapidly in the cells, which led to less cleavage at unwanted loci [86,87]. The RNP strategy has been successfully used in several plants, but in many plants, only *Agrobacterium*-mediated stable transformation can be used [41,88,89]. However, the protoplast transformation has some shortcomings, including hard regeneration and required expensive equipment. Therefore, it is better to apply alternative methods such as nanocarriers. Due to the potential of RNP to decrease unwanted changes, we suggest a nanoparticle-mediated RNP delivery system into intact plants. The recommended system is species-independent, affordable, time-effective, and equipment-independent.

### 3.4. Different Cas Variants

Two conventional genome editing tools in plants are the CRISPR/Cas9 and Cas12a (Cpf1) systems [39]. The major differences between these two nucleases are as follows: First, Cas12a and Cas9 recognize TTTV (V=A, C or G) and NGG PAMs, respectively, and second, Cas12a requires only a crRNA (without tracrRNA). Lee et al. (2018) investigated the efficiency of these nucleases in maize plants. To compare Cas9 and Cpf1, they used the *glossary2* (*gl2*) gene, which contains the recognition sequence for both nucleases. Their results indicate that the CRISPR/Cas9 system is more efficient and specific than Cpf1 (90–100% vs. 0–60% on-target mutations in T0 plants for Cas9 and Cpf1, respectively) [39]. The efficiency of Cpf1 is highly dependent on the crRNA sequence, and crRNAs with a stable secondary structure in the targeting region have lower efficiency [90,91]. The sensitivity to a secondary structure in the Cas12a crRNA is higher than in the Cas9 gRNA [91]. There are other natural variants of Cas9, called SaCas9 (*Staphylococcus aureus*) and StCas9 (*Streptococcus thermophilus*), that recognize longer PAM sequences than SpCas9 (*Streptococcus pyogenes*), including NNGRRT and NNAGAAW [92,93]. Wolter et al. (2018) revealed that by using SaCas9, much higher on-target mutagenesis compared to the use of SpCas9 could be obtained in *A. thaliana*. Therefore, they can also be utilized to increase specific mutagenesis and decrease off-target changes. Two altered versions of SpCas9, named SpCas9-VQR and SpCas9-EQR, have been applied to recognize atypical targets in *A. thaliana* [94]. SpCas9-VQR and SpCas9-EQR recognize NGAN, or NGNG and NGAG, as PAM sequences, respectively. Based on a study by Anders et al. (2016), the use of altered versions of SpCas9 can lead to stabilization of the bindings between the Cas9 and the targeted DNA [95]. Yamato et al. (2019) revealed the ability of SpCas9-VQR and SpCas9-EQR to knock-out *CLAVATA 3* and *ASYMMETRIC LEAVES 1* genes. According to their results, more sgRNAs could be designed using the SpCas9 variants compared to the conventional SpCas9; thus, this system has demonstrated sufficient ability to edit *A. thaliana* genome and could be effective in recognizing atypical targets. One of the conventional methods to suppress off-target effects in plants and mammals is using a Cas9-paired nickase that contains a mutation in one of the nuclease domains (HNH or RuvC-like). The decreased frequency of on-target mutations is one of the shortcomings of the mentioned enzyme [64]. The 3′-overhang created by the Cas9-paired nickase has been shown to result in a lower frequency of on-target mutation than a 5′-overhang structure, which could be related to the 3′-overhang potential as a trigger for the initial step of the HR repair mechanism [64]. Generally, the main advantage of the Cas9-paired nickase compared the Cas9 is its potential to decrease unwanted mutations. Recently, a nuclease-deactivated variant of Cas9 (dCas9) has been widely used in the CRISPR genome editing system to avoid off-target mutations [96]. Mutations in the nuclease domains of Cas9 result in dCas9, which can bind to the target sequence and block transcription elongation [97]. The dCas9 mutant fused to the cytidine deaminase enzyme has been used in several studies that have indicated its ability to convert C to T and G to A in the target plant gene [98,99,100]. The CRISPR/dCas9 can be used as a platform to regulate gene expression in plants, as well. Transcription effectors such as VP64 and SRDX can be fused to dCas9 and lead to activation or deactivation of the targeted gene, respectively [101]. Piatek et al. (2015) examined the ability of the dCas9 fused to EDLL (transcription activator) and SRDX (transcription repressor) to regulate transcription of the target gene. Their data showed that the CRISPR/dCas9-activator or -repressor can be used for targeted gene regulation in plants [102]. In a study by Wyvekens et al. (2015), two strategies for reducing unintended mutations have been utilized: Truncated gRNA (tru-gRNA) and dimerization dependent on the RNA guide FokI-dCas9 nuclease (RFN) [103]. The tru-gRNAs have lengths of less than 20 bp and, by adding two guanidine residues at the 5′ end of the sequence, can be used to decrease off-target effects while maintaining the on-target activity [25,71]. The RFN is created by binding the dimerization-dependent nonspecific FokI cleavage domain to the amino-terminus of the dCas9 [104]. Based on the findings of Wyvekens et al. (2015), the tru-RFN system demonstrated a high frequency of on-target mutations and a low frequency of the off-target mutations in human cancer cell lines. Therefore, this method can also be used in plants to improve the specificity of CRISPR.

### 3.5. Aptazyme Overcomes CRISPR/Cas9 Limitations

**Figure 1:**
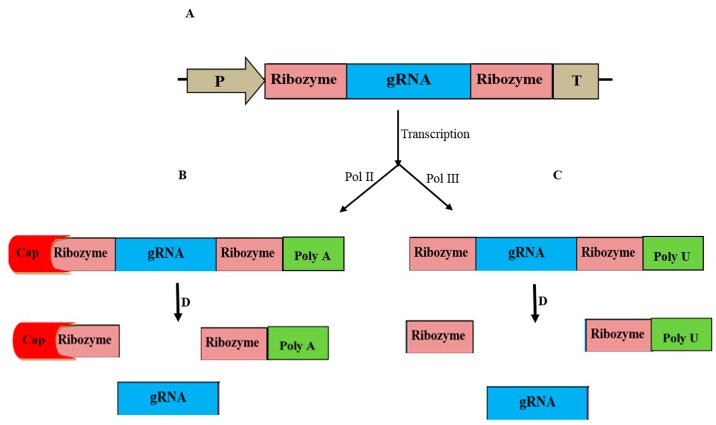
Schematic diagram of the ribozyme-flanked artificial sgRNA (RGR). (**A**) An RGR construction (P: Any promoter; T: Terminator; Ribozyme: Self-cleaving ribozyme; and sgRNA: Single guide RNA); (**B**) Transcription of the sgRNA by RNA polymerase II (Pol II) causes changes in the primary transcript, including 5′-end capping and 3′-end poly A tail; (**C**) Transcription of the sgRNA by RNA polymerase III (Pol III) causes changes in the primary transcript, including 3′-end poly U tail; and (**D**) Ribozymes cleave the 5′-end and 3′-end modifications and produce the unchanged sgRNAs.

The CRISPR/Cas9 system has other limitations that may impact its efficiency, including limited promoters suitable for the in vivo production of sgRNA and the lack of a suitable method for detecting Cas9-free stable plant mutants [105]. RNA polymerase II (Pol II) is not sufficient to produce gRNA in vivo because modification of the primary sgRNA transcripts, including 5′-capping and 3′-polyadenylation, decreases the efficiency of sgRNA and genome editing. Generally, in vivo sgRNA production occurs using RNA polymerase III (Pol III) [26]. U3 and U6 promoters have been successfully used to generate sgRNA molecules in various plants [33,69,89,106,107]. However, the U3 and U6 promoters have some disadvantages, including constitutive expression of the sgRNA, and the initiation of transcription requires the presence of G and A nucleotides at the start of the target sequences for the U6 and U3 promoters, respectively. In addition, the primary transcripts of these promoters have a poly-U tail that may reduce the efficiency of genome editing [108]. Therefore, introducing new technology to prevent sgRNA molecule modification may increase the efficiency of the CRISPR system [109]. He et al. (2017) introduced a ribozyme-based sgRNA strategy to overcome these limitations. They designed a ribozyme-flanked artificial sgRNA (RGR). The RGR can be used under the control of conventional promoters due to its nuclease activity, which catalyzes the cleavage of any modifications, such as polyadenylation [65,110]. It is necessary to follow some points in designing RGR: (1) The cleavage site of the 5′-end ribozyme and the 3′-end ribozyme should be before the first nucleotide and after the last nucleotide of the sgRNA, respectively [105]; and (2) the characteristics of the ribozyme are important in the RGR strategy. A ribozyme with properties such as small size and flank sequence-independent cleavage activity can be used. [105]. A hammerhead ribozyme less than 50 bp in length and containing 6 bp reverse complementary to the first 6 bp of the sgRNA can be used at the 5′-end of the RGR. The mentioned ribozyme may also be used at the 3′-end of the RGR (Figure 1) [105].

**Figure 2:**
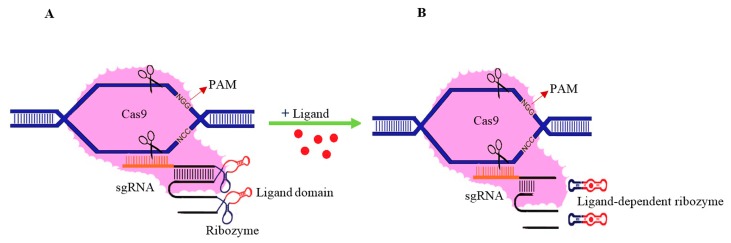
Schematic diagram of sgNA/ligand-dependent ribozymes. (**A**) The ligand-dependent ribozymes (AZ) are inserted into the sgRNA stem-loop structures; and (**B**) in the presence of the ligand, the AZ cleaves itself which leads to the sgRNA degradation.

More than 30 years ago, some small RNA molecules were discovered and called ribozymes [111]. A ribozyme acts as a molecular scaffold to sequester the ribosome binding site (RBS). It can be helpful in genome editing. The hammerhead ribozyme consists of an intramolecular helix (helix II) and two intermolecular helices (helix I and III). The single-stranded regions are completely conserved and contain specific nucleotides for optimum catalytic activity [112]. Some of the ribozymes are ligand-dependent and help scientists control the expression of the target genes [113]. First, Tang and Breaker (1997) fused the ATP-binding aptamer to stem-loop II of the HHR, which led to the design of an allosteric ribozyme called an aptazyme [114]. Saragliadis et al. (2012) attached a theophylline aptamer to stem III of the HHR and obtained an artificial theophylline-dependent aptazyme [115]. Chen et al. (2018) introduced the most effective method to decrease off-target mutations using an aptazyme (AZ; a self-cleaving ribozyme) [116]. They inserted a theophylline-dependent aptazyme into the backbone of the Cas9/sgRNA construction, instead of the tetraloop to control Cas9 expression in the presence of theophylline. Based on the results of Konermann et al. (2015), the tetraloop and stem-loop II of the sgRNA can tolerate aptamer addition with no effect on the sgRNA activity [117]. In this method, posttranscriptional regulation has been used for the activity of the sgRNA. Inserting AZ into both the tetraloop and the stem-loop II of the sgRNA can achieve greater efficiency in controlling the sgRNA activity than inserting AZ only into the tetraloop or stem-loop II of the sgRNA [116]. The mentioned insertion did not affect the on-target mutation, and adding theophylline to the cell culture after 48 h led to cleavage of the sgRNA and the termination of genome editing. This approach is simple and reliable, and might also be helpful to decrease the off-target effects in plants. According to functional studies, the repeat-antirepeat duplex and stem-loop I of Cas9 are necessary for Cas9-sgRNA complex formation, whereas stem-loop II and stem-loop III are not essential for function [118]. Therefore, the aptazyme can be attached to stem-loops II and III (Figure 2). The abovementioned approach can be used to decrease the unwanted mutations of the CRISPR/Cas9 system by attaching a suitable aptamer to the HHR and designing an efficient aptazyme. Theophylline occurs naturally in plants; therefore, the theophylline-dependent aptazyme cannot be used in plants. Further research is needed to design an efficient aptazyme with a ligand that does not naturally occur in plants.

### 3.6. Temperature Effects on On- and Off-Targets

Recently, the effect of temperature as a factor to increase target mutagenesis has been considered. LeBlanc et al. (2018) revealed that the temperature of the growth chamber can increase on-target mutagenesis in *A. thaliana* plants (containing a sgRNA against reporter gene), followed by decreasing of off-targets. Their results demonstrated that plants exposed to heat stress (37 °C) show higher on-target mutations by CRISPR/Cas9 than plants exposed to standard temperature (22 °C) [29]. They observed only 12% GFP-positive in exposing plants to heat stress, while it was 89% in plants exposed to 22 °C. The difference in mutation rate may be due to the high activity of SpCas9 at 32 °C compared to its activity at 22 °C, which is closer to the optimal growth temperature of *S. pyogenes* (40 °C) [29]. For more confirmation, they also investigated the importance of temperature in Cas9 activity in *C. sinensis*. They used plants containing an sgRNA targeting the *Citrus phytoene desaturase* (*CsPDS*) gene that led to albino phenotype. Based on their results, plants subjected to 37 °C revealed enhanced albino phenotype, while exposing plants to 24 °C did not illustrate enhanced white-colored phenotype. Therefore, exposure to 37 °C affects Cas9 cleavage activity and increases on-target mutagenesis in both *A. thaliana* and *C. sinensis*. Their results are consistent with those of Xiang et al. (2017) in human cells [119]. Based on these results, heat stress can be used in other plants that are typically grown at 22–24 °C to achieve maximum on-target mutagenesis. Finding new Cas9 variants or using genetic engineering to produce new Cas9 with optimal activity at 22–24 °C might be helpful to increase on-target mutagenesis in organisms [29]. Based on the results of Lee et al. (2018), Moreno-Mateos et al. (2017), and LeBlanc et al. (2018), the efficiency of Cas9 and Cpf1 is strongly dependent on temperature, and they have higher activities at 37 and 34 °C, respectively, than at 28 °C [29,39,120]. Low temperature (20–28 °C) is commonly used in *Agrobacterium*-mediated transformation; thus, the efficiency of Cas9 and Cpf1 decreases during transformation. Therefore, the use of other temperature-independent transformation methods is preferable.

## 4. Preassembled sgRNAs Cause Savings to Time and Cost

For breeding projects, the production of transgene-free plants is more acceptable to the public. A traditional method for detecting Cas9-free plants is PCR analysis, but the time and cost to screen a large population are high. Thus, the use of a transgene-free plant identification at the seed stage is useful. Recently, Tang et al. (2018) introduced a new CRISPR/Cas9 vector called pKSE401G, which can be helpful in identifying Cas9-free mutated plants in a large population. This vector contains two sgRNAs and a GFP reporter marker; thus, transgene-free plants can easily be detected in the T2 generation. The pKSE401G vector has some advantages, including saving time and cost. However, gene silencing may result in the absence of the GFP signal; that is, T-DNA insertion occurs in the genome. Therefore, other screening methods, such as PCR amplification using transgene-specific primers, should be combined with fluorescent signal screening to select transgene-free plants accurately [121]. One method to produce transgene-free mutated plants is the delivery of preassembled sgRNA and nuclease complexes into plant cells through particle bombardment or protoplast transfection [89]. The selection of an appropriate method to increase on-target mutation is necessary. There are several methods for transferring the construct into plants, such as particle bombardment [122,123], transfer to protoplasts [124], and *Agrobacterium*-mediated [125] and nanoparticle-mediated transformation [4,126], which have certain disadvantages. Among these methods, nanoparticle-mediated transformation is the most effective, simple, and time- and cost-effective approach [126].

## 5. Conclusions

**Figure 3:**
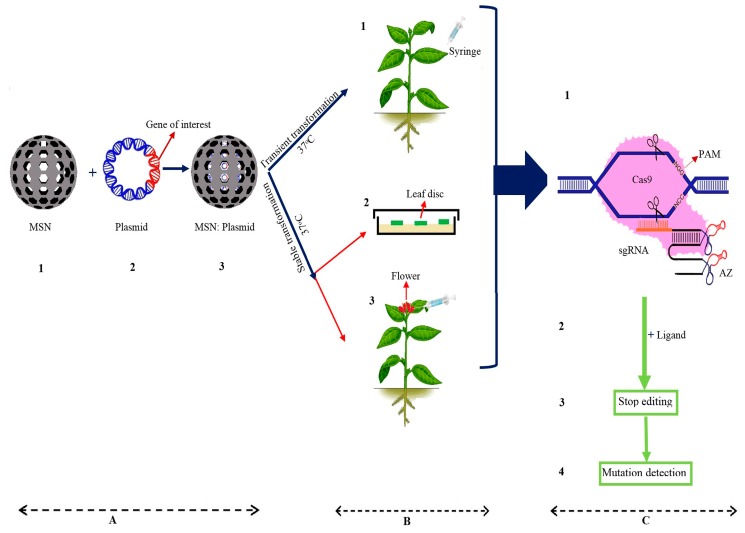
Schematic diagram of the best combination method to avoid off-target mutations in plants. (**A1**,**A2**) Positive charged mesoporous silica nanoparticles (MSNs) and plasmid (pDNA), respectively; (**A3**) the MSNs containing the pDNA; (**B1**–**B3**) three different pDNA-MSNs transient (injection pDNA-MSNs complex into the abaxial surface of leaf) and stable transformation (plant tissue culture and injection pDNA-MSNs complex into the ovary of flower) methods under the temperature of 37 °C, respectively; (**C1**) sgRNA-aptazyme (AZ) finds a homologous target gene and Cas9 cleaves the target; (**C2**,**C3**) in the presence of the ligand, the AZ cleaves itself that leads to the sgRNA degradation; and (**C4**) finally, using several molecular techniques for detection the on-target and the off-target mutations.

Taken together, the evidence shows that CRISPR/Cas9 is a powerful and precise tool for genome editing in many organisms. The main concern about the CRISPR system is off-target mutations. In this paper, various approaches to decrease the off-target effects have been discussed. The efficiency of the CRISPR/Cas9 system depends on the features of the target site, the design of the sgRNA, the properties of the endonuclease, the Cas9 or Cpf1/sgRNA complex concentration, and the transformation method. According to this review, a combination method can be used to increase on-target mutagenesis and to reduce the frequency of unintended mutations. On the other hand, the use of alternative temperature-independent methods, such as mesoporous silica nanoparticle (MSN)-mediated gene transformation, can be helpful. Based on human research, the use of Cas9/sgRNA-AZ leads to the avoidance of off-target mutations in human cells; therefore, we suggest using MSN:Cas9/sgRNA-ribozyme at a transformation temperature of 34–37 °C to strongly decrease unwanted mutations (Figure 3). These findings provide a new, simple, and fast gene transformation and genome editing approach with advantages including reduced time and energy consumption, the avoidance of unwanted mutations, increased frequency of on-target changes, and no need for external forces or expensive equipment.

## Figures and Tables

**Table 1:** List of some recent CRISPR studies in different plants considering the transformation method.

Plant Species	sgRNA Promoter	Cas9 or Cpf1 Promoter	Off-Target Mutation (%)	On-Target Effects (%)	Transformation Method/Off-Target Decreasing Strategy	Reference
*Arabidopsis thaliana*	AtU6	35DPPDK	Not detected	1.1–5.6	Protoplast transfection/highly specific sgRNA	[26]
	AtU6	AtRPS5a	<0.1	85	*Agrobacterium*-mediated transformation/appropriate promoter	[27]
	AtU6	CaMV35S	N/A	6.5	Protoplast transfection/-	[28]
	AtU6	AtYAO	Not detected	86	*Agrobacterium*-mediated transformation/heat stress	[29]
	AtU626	2 X CaMV35S	Not detected	24	*Agrobacterium*-mediated transformation/dual sgRNAs-Cas9 vector	[30]
*Oryza sativa*	OsU3	CaMV35S	N/A	7.3	Protoplast transfection/-	[28]
	OsU6	2 X CaMV35S	Not detected	8	Particle bombardment transformation/Cpf1	[31]
	OsU3	CaMV35S	Not detected	5	Particle bombardment transformation/highly specific sgRNA	[32]
	OsU3	ZmUbi	0–2.2	85	*Agrobacterium*-mediated transformation/careful sgRNA design	[33]
	OsU3	ZmUbi	N/A	90.6	Particle bombardment transformation/ dual sgRNAs-Cas9 vector	[34]
	OsU3	ZmUbi	N/A	75	*Agrobacterium*-mediated transformation/dual sgRNAs-Cas9 vector	[34]
*Brassica napus*	AtU6	Ubi	Not detected	5.3–100	*Agrobacterium*-mediated transformation/careful sgRNA design	[35]
	AtU6-26 AtU3b AtU6-1	CaMV35S	N/A	0–54.5	*Agrobacterium*-mediated transformation/efficiency depends on the targeting specificity of sgRNA	[36]
*Brassica oleracea*	-	-	Not detected	0.09–2.25	Protoplast transfection/RNPs	[37]
	AtU6-26	PcUbi4-2	Not detected	100	*Agrobacterium*-mediated transformation/-	[38]
*Zea maize*	OsU3	ZmUbi	Not detected	90–100	*Agrobacterium*-mediated transformation/highly specific sgRNA	[39]
	ZmU6	ZmUbi	Not detected	10.67	*Agrobacterium*-mediated transformation/highly specific sgRNA	[40]
	-	-	Not detected	47	Protoplast transfection/RNPs	[41]
	ZmU3	ZmUbi	Not detected	10	*Agrobacterium*-mediated transformation/Cas9 nickase- cytidine deaminase fusion	[42]
*Triticum aestivum*	TaU3 TaU6 OsU3	Rice Actin	Not detected	11–17	*Agrobacterium*-mediated transformation/-	[43]
	CaMV35S	CaMV35S	Not detected	18–22	*Agrobacterium*-mediated transformation/careful sgRNA design	[17]
	-	-	5.7	21.8–33.4	Protoplast transfection/RNPs	[44]
	TaU6	ZmUbi	Not detected	1	Particle bombardment transformation/Cas9 nickase- cytidine deaminase fusion	[42]
*Vitis vinifera*	AtU3b AtU6-1 AtU6-29	2 X CaMV35S	Not detected	31	*Agrobacterium*-mediated transformation/-	[45]
	-	-	Not detected	0.1	Protoplast transfection/RNPs	[46]
	AtU6	CaMV35S	Not detected	Up to 100	*Agrobacterium*-mediated transformation/careful sgRNA design	[47]
*Solanum lycopersicum*	AtU6-26	SIUbi10 PcUbi4	N/A	Up to 90	*Agrobacterium*-mediated transformation/using geminiviral replicon	[48]
	AtU6-26	2 X CaMV35S PcUbi4-2	Not detected	Up to 100	*Agrobacterium*-mediated transformation/designing highly specific sgRNAs	[49]
	AtU6-26	PcUbi4-2	Not detected	Up to 71	*Agrobacterium*-mediated transformation/using CRISPR cytidine base editors	[50]
	AtU6	2 X CaMV35S AtUbi	Not detected	72.7–100	*Agrobacterium*-mediated transformation/three sgRNAs with a GC content >50%	[51]
	AtU6	2xCaMV35S	N/A	75–100	*Agrobacterium*-mediated transformation/dual sgRNAs-Cas9 vector	[52]
*Malus prunifolia*	-	-	Not detected	0.5–6.9	Protoplast transfection/RNPs	[46]
	AtU6-1	2 X CaMV35S	Not detected	31.8	*Agrobacterium*-mediated transformation/truncated sgRNAs	[53]
*Nicotiana benthamiana*	AtU6	35DPPDK	Not detected	37.7–38.5	Protoplast transfection/highly specific sgRNA	[26]
	AtU6	CaMV35S	Not detected	1.8–2.4	*Agrobacterium*-mediated transformation/careful sgRNA design	[54]
	CaMV35S	CaMV35S	Not detected	0–12.7	*Agrobacterium*-mediated transformation/careful sgRNA design	[17]
*Populus tremula*	AtU3b AtU3d AtU6-1 At-U6-29	CaMV35S	N/A	51.7	*Agrobacterium*-mediated transformation/-	[55]
	MtU6-6	CaMV35S	Not detected	100	*Agrobacterium*-mediated transformation/careful sgRNA design	[56]
*Medicago sativa*	AtU6	Ubi	Not detected	2.2	*Agrobacterium*-mediated transformation/ highly specific sgRNA	[57]
*Medicago tranculata*	MtU6	2 X CaMV35S	Not detected	10.4	*Agrobacterium*-mediated transformation/highly specific sgRNA	[58]
*Theobroma cacao*	AtU6-26	CaMV35S	Not detected	27	*Agrobacterium*-mediated transformation/-	[59]

-: No special method has been used to decrease off-target effects; N/A: Not available; RNP: CRISPR/Cas9 or CRISPR/Cpf1 ribonucleoproteins.

## Abbreviations

CRISPR/Cas9	Clustered regularly interspaced short palindromic repeat/CRISPR associated protein 9
sgRNA	Single guide RNA
crRNA	CRISPR RNA
PAM	Protospacer adjacent motif
NHEJ	Nonhomologous end joining
HDR	Homology-directed repair
ZFNs	Zinc finger nucleases
TALENs	Transcription activator-like effector nucleases
WGS	Whole genome sequencing
RNP	CRISPR/Cas9 or CRISPR/Cpf1 ribonucleoproteins
tru-gRNA	Truncated gRNA
RFN	RNA guide FokI-dCas9 nuclease
RGR	Ribozyme-flanked artificial sgRNA
MSN	Mesoporous silica nanoparticle
